# COVID-19: challenges faced by Nepalese migrants living in Japan

**DOI:** 10.1186/s12889-021-10796-8

**Published:** 2021-04-19

**Authors:** Divya Bhandari, Yasuhiro Kotera, Akihiko Ozaki, Sudeepa Abeysinghe, Makoto Kosaka, Tetsuya Tanimoto

**Affiliations:** 1 Medical Governance Research Institute, Tokyo, Japan; 2 Human Sciences Research Centre, University of Derby, Derby, UK; 3 Department of Breast Surgery, Jyoban Hospital of Tokiwa Foundation, Iwaki, Japan; 4 Global Health Policy Unit, School of Social & Political Science, University of Edinburgh, Edinburgh, UK

**Keywords:** COVID-19, Migrants, Nepalese, Japan, Financial hardship, migrant welfare, mental health, migrant health

## Abstract

**Background:**

Worldwide, COVID-19 has exacerbated the vulnerability of migrants, impacting many facets of their lives. Nepalese make up one of the largest groups of migrants residing in Japan. Crises, such as the ongoing COVID-19 pandemic could disproportionately affect migrants from low- and middle-income countries like Nepal, widening health and economic inequalities. An in-depth, comprehensive assessment is needed to appraise the diverse problems they encounter. Drawing upon qualitative interviews, this study aimed to identify challenges faced by Nepalese migrants in Japan as a consequence of the COVID-19 pandemic and to discuss their needs to counter these challenges.

**Methods:**

This qualitative study employed an interpretivist approach to appraise the first-hand experience of Nepalese migrants living in Japan. Fourteen participants (8 males and 6 females, aged 21 to 47 years old) were recruited to participate in semi-structured in-depth telephone interviews (45–60 min) regarding: (a) their perceived current physical and mental health, (b) problems faced as a result of the COVID-19 pandemic, and (c) perception of available and necessary support structures. Purposive and snowball sampling techniques were used to recruit the participants. Interviews were recorded, transcribed, and thematically analyzed.

**Results:**

Six themes were identified: 1) experiencing psychosomatic symptoms, 2) adoption of new healthy behaviors, 3) financial hardship, 4) family concerns, 5) reflections on discrimination and 6) reflections of existing support and expectations of support systems. The findings of our study illustrate the specific impact of COVID-19 among Nepalese migrants regarding their unstable employment conditions, perceived lack of social support, possible obligation to send money home, difficulty in accessing services due to the language barrier, and a lack of effective governmental support from Nepal. Pandemic-related adversity has negatively impacted migrants’ mental well-being, exacerbating their vulnerability.

**Conclusions:**

Comprehensive and timely support should be provided to the vulnerable migrant population. Effective coordination among relevant parties in both countries, including the governments concerned, should be facilitated.

**Supplementary Information:**

The online version contains supplementary material available at 10.1186/s12889-021-10796-8.

## Background

Although it is widely recognized that migrant workers are major contributors to social and economic development, they often encounter circumstances in which their health and socio-economic well-being are compromised [1, 2]. While new coronavirus (SARS-CoV-2) poses dangers for the general population, evidence indicates that it is taking a particularly heavy toll on vulnerable minority populations [3–6], including individuals who relocate themselves from their home nation to another in search of better opportunities in terms of education and living conditions. Among other factors, living and working conditions [7], cultural and language barriers [8, 9], limited local knowledge, poor social networks [10], and xenophobia [10], all limit the ability of migrant populations to avoid infection, receive adequate health care, and cope with the economic, social, and psychological impacts of the pandemic [11]. They are likely to encounter a double burden related to the risk of infection and the lack of resources when they become infected. For example, in Saudi Arabia, 75% of all new confirmed cases of SARS-CoV-2 infection as of 7 May, and over 95% of the confirmed cases in Singapore by 19 June, were among migrants workers [12, 13]. Migrant workers represent one in five workers in Northern America and Europe, both heavily impacted by COVID-19 [14]. Similarly, 79 and 38% of the total population in Qatar and Saudi Arabia respectively are migrants workers and most of them are working in healthcare, a high-risk sector for infection [15]. While tens of millions of migrant workers have lost their jobs due to the pandemic, many remain trapped in the host countries without housing and/or income [16]. A decrease in income has led to rising debts and insecurity, for families in the home country for whom the remittance from relatives working abroad represent a critical income source of income [17]. Furthermore, preventive behavior, such as physical distancing, may not be possible for migrants living in crowded living conditions, making them even more at risk of SARS-CoV-2 infection [18].

In addition to the direct impacts of transmission, attitudes towards migrants are not always neutral or positive and the case of stigmatization and discrimination in crises in relation to infectious disease outbreaks is regularly evident (e.g., migrants were negatively perceived and treated during cholera in the 1830s, HIV/ AIDS in the 1980s or, more recently, with regard to H1N1 influenza) [19–22]. With the spread of COVID-19 globally, there has been an increased reportage of verbal abuse and stigmatization against certain nationalities [23–25], as seen in anti-Semitic conspiracy theories and COVID-19-related anti-Muslim attacks in different countries [26]. Stigmatization and social marginalization can exacerbate infectious disease transmission and impede healthcare through multiple mechanisms. This includes the exacerbation of acts of concealing symptoms [27, 28], social and physical isolation, and worsening of precarious economic circumstances [29].

Like many other countries, Japan is a key regional destination for migrant workers. The number of migrants in Japan has almost tripled from the year 1990 reaching 2.82 million (2.22% of the total population) in 2019 [30]. With a ‘super-aged’ population (where more than 20% of the population is aged 65 years and older), Japan is facing demographic challenges resulting in important societal and economic consequences [31]. Over 85% of the migrant workers in Japan are of working age (15–65 years) and they play a significant role in maintaining economic stability amid a chronic labor shortage [30]. Despite the Japanese government’s effort to establish a migrant-friendly environment [32], some kind of discrimination and stigma still exists. A survey conducted by Japan’s Ministry of Justice among 18,500 migrants living in Japan revealed that 30% had experienced discrimination, 40% were rejected for rent by landlords, and 25% were denied a job on the basis of their nationality [33]. The new reality of the COVID-19 pandemic is more likely to affect the life of migrants in Japan, magnifying these existing processes of social marginalization [33].

In Japan, the confirmed cases of COVID-19 were > 140,000 as of 28 November with 2028 confirmed deaths [34]. Despite the government’s emergency declaration in April, and significant precautionary measures to safeguard against the COVID-19 outbreak, to date the country has not undergone a complete lockdown. Japan has faced numerous setbacks in 2020 (e.g. postponement of the Olympic Games, typhoon Haishen hitting major cities), which have directly impacted the nation’s economy [35]. Furthermore, COVID-19 has negatively affected an already weakened economy by impacting businesses at home and abroad. A recent survey conducted by Japan’s Ministry of Internal Affairs and Communications revealed that 2.10 million people in Japan were unemployed in September 2020, with an increase of 420,000 from the previous year [36]. In such circumstances, foreign workers are particularly vulnerable and most likely to be made redundant first [37].

Furthermore, these impacts are compounded for the millions of migrants from low- and middle-income countries like Nepal, which widens health and economic inequalities surrounding ethnicity and migrant status [16]. Among foreign workers in Japan by nationalities, Nepalese are one of the largest groups. The rate of migration from Nepal has seen a more than 7-fold increase over the last decade, reaching a total of 96,824 by the end of 2019. The Nepalese officially resident in Japan include dependents (29,992), students (29,417), skilled labor (12,679), and other residential statuses [30]. Nepalese pay significantly towards educational consultancies, restaurant owners, or local agents and recruitment agents in Nepal [38], to enter the country as language school students and to work as cooks in Japan [39]. Some migrants put their land and property at stake to borrow the heavy debt needed to enter and work in Japan [39]. Students and dependents are allowed to work for up to 28 h per week and there is no language proficiency test as a prerequisite for their entry; this makes Japan an alluring option for such migrant categories [31]. The average wage in Japan, which is more than 10 fold higher than in Nepal [40], can serve to motivate Nepalese individuals to migrate and seek out a higher standard of living. However, to compensate for the cost of living and tuition fees, often such individuals end up working more than the legally permitted work limit, within the informal economy [35]. Long working hours, decreased access to health information, and unhealthy living circumstances make them prone to physical and mental ill-health [41].

Mental health is becoming a growing problem among international migrants living in Japan. While counseling is not commonly included in health insurance in Japan, the language barrier is another hindrance that prevents migrants from seeking those services [42]. Some migrants do not hold /renew their health insurance, which prevents them from seeking timely health care and support [41]. It is important for everyone to better understand the challenges faced by the non-Japanese individuals living in Japan and identify potential interventions to improve their overall health and wellbeing, especially with respect to the COVID-19 pandemic. Therefore, this study aims to: a) identify challenges faced by Nepalese migrants living in Japan during the COVID-19 pandemic, and b) explore what assistance they need to overcome these challenges.

The study addresses these questions by focusing on the reported lived experiences of members of the migrant community, interpreted and analyzed through a qualitative approach.

## Methods

### Study design and participants

Qualitative in-depth telephone interviews were conducted with Nepalese migrants living in two urban centers in Japan. An interpretivist approach was used to determine the experiences of health and wellbeing through the perspectives of those participants in the study [43]. An interpretivist approach seeks to understand the social world through the perspectives of research participants, showing how these experiences are embedded within wider socio-political structures [44]. The inclusion criteria for the recruitment of participants were 1) at least 18 years of age, 2) having lived in Japan for at least 6 months, 3) having no diagnosed mental health problems (due to issues of distress during the study period). Purposive and snowball sampling techniques were used to recruit participants. Firstly, the lead researcher DB approached the participants, community leaders within the Nepalese migrant community known through her contacts. These ‘gatekeepers’ identified Nepalese participants, and then snowballing from these connections was used to recruit further participants. The gatekeepers did not participate in the study, so all participants were not previously personally and professionally known to DB, who was introduced to them as a Nepalese citizen working as a public health researcher in Tokyo. Purposive sampling was selected to increase the heterogeneity of the sample, differing in age, gender, and educational level, to better understand different perspectives and problems. Snowballing from direct contacts was used to extend the participant pool, again paying attention to the diversity of the overall sample [45]. In total, 16 migrants were approached for the study, and 14 agreed and completed the interview (5 from the initial direct contact, and 9 from snowball sampling). The two who did not participate were not asked for a reason for non-participation as per study ethics. After the 14th interview, all researchers agreed that more interviews would not offer new findings to confirm data saturation, thus recruitment was completed.

### Interview guide

The semi-structured interview guide was developed by a group of five researchers from multiple disciplines involved in this study (public health researcher, social scientist, psychologist, and medical doctors) after extensive reviewing of the extant literature and following a detailed discussion around the sampling strategy and participant characteristics. The English language version of the interview guide was translated into Nepali by DB (who is a native Nepali speaker). Two Nepalese independent translators were also enlisted to review the wording and translation of the interview guide to ensure effective communication with the participants. The interview guide was piloted on two Nepalese migrants living in Japan before conducting the main interviews. Those two people were approached conveniently for a pilot test through the researcher’s own personal contacts. No major sources of incomprehensibility were noted following the pilot testing. The interview guide developed and used in this study is included as an ‘Additional file 1’.

The interview guide examined three main questions 1) asking participants about their self-perceived current physical and mental health; 2) eliciting reflections around the challenges faced during COVID-19; 3) understanding perceptions around support and perceived support needs. Furthermore, socio-demographic variables including age, gender, length of time in Japan, visa status, employment status, financial status, education level, and Japanese language skills were also collected.

### Data collection

Data were collected from June 15 to July 9, 2020. The purpose and procedure of the study were described in detail to participants before scheduling an interview at their convenience. Qualitative in-depth interviews were conducted via telephone. Before starting the interviews, participants were informed about i) the study objectives, ii) the voluntary nature of participation, iii) how the data will be managed/disseminated and iv) the researcher’s contact information. Both verbal and written consents were collected before starting the formal interview. The participants were informed that they were able to withdraw their responses before the authors finalize their manuscripts. The study participants were able to withdraw from the study at any time without providing a reason for withdrawal. Interviews were conducted in the Nepali language, as the native language of the participants and the lead researcher (DB). The participants were given enough time to express their opinion. Each interview lasted for around 45–60 min and was audio-recorded with the permission of the participants. A comfortable environment was created using techniques such as active listening, open-ended questioning, and clarification to maintain the authenticity of the data and to avoid response bias [46]. Each recording was transcribed by DB, who (also a Nepalese citizen) has experience conducting educational programs, focus group discussions, and in-depth interviews among Nepalese residents in Japan. Recordings and the transcribed data were password-protected and were stored on DB’s password-protected computer. Confidentiality was maintained by coding the audio and transcripts in numbers (e.g., participant P1, P2, etc.) and by removing identifiable information from the transcripts prior to the start of the analysis.

### Data analysis

Thematic analysis was chosen in light of the nature of the data and the research task [47]. Translated semi-structured interview data was collected which is exploratory and explanatory, based on the limited evidence that exists about this research question and the specific population group in Japan.

Analysis procedure included the following process:

Thematic data analysis followed the six stages recommended by Braun and Clarke [47]. Initially, the lead researcher read and re-read transcripts in Nepalese, in order to identify patterns of the meaning and produce a broad picture of the data by generating initial codes. There were in total 42 initial codes. The second level of analysis involved reviewing the codes, which were translated into English. The accuracy of code-generation and the translation of codes were cross-checked by another Nepalese-English bilingual individual. At this stage, the main focus was given to retaining the diversity of the initial codes while producing a higher level of subthemes. Codes with similar meanings were grouped in subthemes according to their differences and similarities [48]. The coding technique and process were checked by all members of the research team. This was particularly essential in this study to avoid interviewer bias, as the interviewer was also a Nepali living in Japan. Next, DB and two co-researchers YK and AO independently identified themes from the data, and a discussion was held to set the agreed themes. Then, all researchers reviewed the themes to agree with the final themes, as reflected in this paper. Analyses were performed manually: no software was used.

## Results

The demographic details of the interviewees are presented in Table 1. As evident from the table, the sample was diverse with respect to age, sex, educational level, duration of stay in Japan as well as visa status. Of the 14 Nepalese migrants who were interviewed, 8 were male and 6 were female. The age range was between 21 and 47 years. Their duration of stay in Japan ranged from 1 to 16 years. Eight were full-time workers, and 6 were part-time workers. Participants experienced a significant decline in their monthly income from before to after the onset of COVID-19. The median monthly income pre-COVID-19 was 202,500 yen (approximately 1900 USD), which was reduced to 80,000 yen (approximately 750 USD) during the course of the COVID-19 pandemic. Though the participant sample was small for a quantitative appraisal, this income reduction was a salient aspect of the research findings in that it provides evidence for the increasing precarity of this population as a result of the pandemic.

**Table 1:** Socio-demographic characteristics of participants

Socio-demographic characteristics	n	%
**Age**		
20–30 years	7	50.0
30–40 years	4	28.6
above 40 years	3	21.4
**Gender**		
Male	8	57.1
Female	6	42.9
**Length of stay in Japan**		
1–5 years	6	42.9
5–10 years	4	28.6
above 10 years	4	28.6
**Visa/Residence status**		
Student	5	35.7
Dependent	1	7.1
Working	5	35.7
Long-term	3	21.4
**Monthly income in JPY**, median [min-max]		
Before COVID-19	202,500 [100,000-600,000]	
Current (During data collection)	80,000 [0–200,000]	
**Employment status**		
Full-time worker	8	57.1
Part-time worker	6	42.9
**Education level**		
Primary and Secondary level	2	14.3
Higher secondary level	6	42.9
Bachelor level	4	28.6
Master’s level or higher	2	14.3
**Japanese language skill**		
Beginner	8	57.1
Intermediate	4	28.6
Advance	2	14.3

*JPY* Japanese Yen

Our analysis revealed challenges encountered by Nepalese citizens residents in Japan in different areas of their life including economic security, family life, mental health, and other related challenges. They offer guidance with regard to how authorities could better support their wellbeing with respect to these areas. Six themes were identified: 1) experiencing self-perceived psychosomatic symptoms, 2) adoption of new healthy behaviors, 3) financial hardship, 4) family concerns, 5) reflections on discrimination, and 6) reflections on existing support and expectations of support system. These themes are presented in the order of dominance within the data. Participants were coded by number and by sex (i.e., M1 = Male participant number 1, F2 = Female participant number 2).

### Theme 1: experiencing psychosomatic symptoms

#### Worried feelings and thoughts

Almost all participants experienced a significant amount of negative emotions such as being worried, afraid, restless, and anxious. Economic problems, family concerns, fear of being exposed to the virus, information overload, lack of social support, and language barriers were major sources of stress among participants in this study.

Economic impacts were one of the key drivers for these experiences:

*F3: … I got a notice that my job contract has been canceled due to COVID. I have been living in the company’s apartment. However, I need to leave this apartment as I no longer have that job. It is very difficult to find a room in Japan unless I have a good job. I am completely clueless and extremely worried as I do not think I will be able to find a stable job in this crisis. I also do not have any friends and relatives living close to me.*

*M10: I used to work as a research assistant until March. My job was not terminated because of COVID. However, I had to search for a new part-time job to pay my tuition fee, and the COVID made the situation difficult for me to find one. My friends were jobless for 3-4 months. We were stressed and had trouble sleeping for 1-2 months as we were unable to pay room rent and tuition fees. We could not go back to our country nor able to sustain our stressful life in Japan.*

These examples demonstrate the link between economic precarity, and the social stress experienced by the participants.

Participants were also worried about being exposed to the virus in their workplace or while commuting. They pointed out how experiencing minor health problems such as headaches and fever caused a sense of insecurity. One of the participants indicated having physical symptoms, like chest pain due to stress.

*F6: When I started having mild symptoms including fever, my entire family and I became stressed. My condition was so severe that, I used to have pain in the right chest radiating to my back while breathing. The doctor told me that it was due to stress and asked me to take a rest and relax. … … No, I did not have any preexisting health problems.*

Other responses reflect the stress caused by their work environments during the pandemic:

*F1: As I work in a convenience store, I have to deal with many customers. I am usually afraid, I might get COVID-19 while interacting with them. Sometimes, I even think, I might die due to COVID-19 and may not be able to see my family again.*

This reinforces the well-known link between economic status and socioeconomic determinants of physical and mental health.

Information overload on social media such as Facebook could escalate stress. Participants noted how too much (unstructured and unverified) information intake added stress in their daily life. In addition, issues around testing for infection with SARS-CoV-2 virus magnified the uncertainties they faced.

*F13: Too much news and information about COVID-19 on online platforms like Facebook made me worried. As I am living in a foreign country, I am worried about thinking, ‘what if I get infected who will take care of me? How will I be able to manage those situations?’. A sense of insecurity is there, especially living away from family in a foreign nation.*

A lack of social support and access to a reverse transcriptase-polymerase chain reaction (RT-PCR) test was another reason for their stress. Furthermore, limited language skills compounded this effect preventing them from utilizing the available facilities provided by the government.

*F6: I used to have symptoms similar to COVID-19 (101/102 °F fever, cough, etc.). I went to seek treatment and the doctor advised me to self-isolate at home for 14 days. I was desperately seeking for COVID-19 test; however, I was not able to do so. Rather it was an arduous task to gather and understand the information about available facilities. I was not able to sleep for 1-2 months because of stress. I used to spend the whole night listening to music. That was the worst day of my life to date. I was very anxious. Unable to access testing coupled with information overload and misinformation in online media like Facebook made me more vulnerable to depression. I even sought help for counseling.*

*M2: Many Nepalese students living in Japan might be unaware of facilities provided by the Japanese Government amid COVID-19 because of the language problem. I have helped a few Nepalese by sharing information about available facilities and treatment options. I am certain that there are many Nepalese who are having stressful life because of language problems.*

*F6: New Nepalese students coming to Japan might not be competent in Japanese. It is difficult for those students to even seek minor health facilities as most of the information is in Japanese, which makes their life difficult and more stressful.*

These comments highlight the vulnerability of migrants due to the lack of social support, a language barrier, and inadequate information on treatment and testing in connection with the COVID-19 pandemic, which, adversely affected their mental health and wellbeing.

#### Indirect health problems

While most of the participants did not have any physical health problems directly due to infection by COVID-19, they reported indirect health impacts of COVID-19. Some of them reported having minor health problems like “allergies” (believed by respondents to be due to overuse of hand sanitizers), weight gain, gastrointestinal disturbance related to change in dietary habits and exercise patterns, and sleeping difficulty which led to headaches and drowsiness.

*M10: I have started thinking a lot lately (after COVID-19 started) that has negatively affected my sleeping pattern. Due to a lack of sleep, I am having some physical health problems like headaches and drowsiness.*

*F1: I am having an allergy due to the frequent use of hand sanitizers.*

Varying degrees of (possibly psychosomatic) problems were reported by the participants following the onset of the COVID-19 pandemic.

### Theme 2: adoption of new healthy behaviors

Some respondents have adopted new healthy behaviors and perceived themselves as being healthier than before the pandemic began. This is an unexpected result and a significantly recurring theme in the data.

*F13: No, I have not faced any physical problems recently. I have become more conscious regarding my eating and living habits.*

*M9: I and my family are strictly following every safety measure, maybe that's why we do not have any physical health problems lately. Rather, I think we have become healthier than before.*

It seems some participants have embraced proactive steps to maintain a healthier lifestyle, which could in turn have long-term benefits. The participants seemed ready to offer these reflections despite the wider more negative consequences caused by the change of circumstances.

### Theme 3: financial hardship

Almost all participants underwent financial hardship, impacting their work and family lives. Their working hours/shifts were reduced, including some becoming jobless for over 3 months (at the time of data collection). Most respondents had difficulties with sustaining their daily living, which included an inability to pay the rent or tuition, or (for those with businesses) their employees’ salary.

*F4: We (F4 and her husband) have already spent all of our savings. We borrowed money from friends and social circles to pay the rent. I am worried if the situation continues, we will not be able to sustain ourselves here in Japan.*

*F11: I use to work in two places. I was fired from one job and I am relying on the only one [left], whose working shift has also been reduced. I am unable to pay my school fee. There was a complete lockdown in Nepal, so it was difficult for my family to send money here.*

*M9: I am working as a cook in a restaurant. My salary has been reduced by around 50% but expenses are the same. Customers coming to our restaurant have been drastically reduced due to this pandemic. Now, takeout order is only an option for us to manage our business.*

*M8: I have 4 restaurants and shops and employ about 25 workers. I am not able to pay the rent and provide a salary to my staff. My business is completely down.*

*M7: It is depressing to hear that the Nepalese migrants who want to return to Nepal are forced to pay extra money (almost double) for flight tickets to go to Nepal. Most of them want to go back because they are having difficulty sustaining their life in Japan. Some of them do not have money to even afford one flight ticket. It is sad to see that the Nepalese government is not able to help those people in any way.*

The quotes above illustrate the diversity of impacts in relation to lost income, which affect almost all participants but have a more severe impact on those who were previously financially unstable. The cyclical nature of migrant remittances is also evident here, as these participants can be affected by the consequences of COVID-19 on the country of origin in addition to Japan (as seen in the second quote by F11), compounding the negative impacts on these individuals.

### Theme 4: family concerns

Participants’ concerns were not only (or in some cases, not primarily) focused on themselves but also on their wider family. The participants experienced worries about their children’s development. They mentioned that the current situation might have affected their children’s mental health and academic development.

*M7: I have three children and they have not gone to school for around 3 months. Parks were closed and they were not allowed to play outside. I think this negatively impacts children’s mental health.*

*F6: Recently, when I was sick (perceived susceptibility of having COVID-19), I was particularly concerned about my children. I was worried thinking about who will take care of my children if I die.*

*M5: I am more concerned and worried about my children’s education and their future. Their schools are being closed … … Yeah, they have online classes. But, personally, I think classroom interaction is far better than online education for the overall development of children.*

Furthermore, travel restrictions compelled participants to live away from their loved ones. While some could not invite their children and spouse to Japan, others faced difficulties returning their parents to Nepal who came to Japan with a visiting visa.

*M7: One of my Nepalese friends was not able to attend the funeral ceremony (which is considered an important ceremony in Nepal and is usually performed by the son) of his parents due to travel restrictions. As he is only one son, he is very stressed right now.*

*M9: I am having difficulty sending my father back home who came here just to visit me for a month. … My child is in Nepal and I am unable to bring him here because of travel restrictions and we do not have any responsible person to take care of him there.*

Participants were worried as they were unable to send money to their dependent parents/relatives in Nepal and pay off their loans. Furthermore, they were concerned about their family members’ safety and felt regretful about being away from them in this uncertain situation.

*F13: I usually have to send money to my family back in Nepal as my parents are completely dependent on me. Also, I have to pay off my loan which I took while coming to Japan. After COVID-19, I am having difficulties sustaining my life in Japan, I cannot think of sending money there. I am worried about my parents back in Nepal.*

*M8: I am particularly worried about my parents back in Nepal. They are getting old and there is no one to take care of them. I am their single son; so, sometimes I feel sad for not being together with them in this situation.*

The pandemic has reshaped family relationships in unprecedented ways, forcing people to live closer/together and, for some, forcing them to live apart and separated from family members and responsibilities. Particularly, the perceived risks/consequences of COVID-19 in the children’s development and dependent family members’ lives have generated an additional burden on the life of participants.

### Theme 5: reflections on discrimination

None of the participants in the study directly experienced COVID-19 related discrimination or stigma. However, a few of them spoke of secondary knowledge of discrimination, in particular, that their friends had to lose their jobs suddenly without prior notice/information during COVID-19 because, according to their account, they were migrants.

*F3: … If there was another Japanese citizen in her place it would have never happened. In the first place, the company would not have terminated him/her suddenly. Even if they do so, they would give a reason for his/her termination and might help them in further steps.*

*M2: … My friend lost his job due to COVID-19. So, he was unable to pay school fees for a month or two then he was continuously threatened to be sent back to Nepal.*

Moreover, participants highlighted perceived discriminations faced personally or by other migrants (friends/relatives) living in Japan. This includes the information gap between employer and employee, difficulty gaining trust, feeling unappreciated, prioritizing citizens in case of better job/opportunities, difficulties finding accommodation, and difficulties carrying out certain business.

*F3: … for example, If I am working with a Japanese colleague then the employer/boss shares all detailed information with him/her. However, I do not receive such detailed information from the boss directly; instead, I only get such information through third parties.*

*M2: … if the same work is done by Japanese and other foreign guys, they easily rely on Japanese and accept their work, but they seem more suspicious whether they can trust us or not as we are foreigners.*

*M10: Although I do not have any personal experience of being discriminated against, I have heard multiple instances where foreigners were rejected by some hospitals for treatment. One of my close friends also shared an incident where he was refused to be treated (before COVID-19). I am not sure what was the exact reason, and my friend did not explain it to me. However, I think this kind of problem is a result of the language barrier or maybe due to a lack of health insurance.*

Migrants, especially those with no official employment contract, will be the first to be made redundant from work and face additional uncertainty during a crisis.

### Theme 6: reflections of existing support and expectations of support system

Within the interviews participants also reflected in sources of support, both in the sense of those that they can already draw upon and those that they wish might be produced for them. This was both in terms of their own perceptions through their recounting of experiences and prompted by an interviewer question. In relation to formal support structures, our participants did not know about or believe they have access to, any local/nongovernmental organizations that are specialized in helping migrants during COVID-19 or similar public health crises.

*F12: I have not heard about any organization that is helping migrants and listening to our concerns. I have no idea where to approach when I face problems.*

Most participants expressed confusion and anxiety about what they might do if they were to become infected with COVID-19. In fact, a key narrative was distrust in any non-governmental organization; the participants expressed an expectation that such organizations would not help them.

*F6: I think it is foolish to believe the organization and NGO, [that they] help people. There are certain people involved in those organizations who want to be popular. They try to prove they are doing something good by posting on social media like Facebook, but everything is a strategy to show and no one is here to selflessly help people in need.*

The majority of participants noted incentives provided by the Japanese government to all residents irrespective of their migration status and expressed their gratitude to the Japanese government. The Japanese government has allocated virus relief fund and provided various facilities for all residents irrespective of migration status such as 100,000 yen (approximately USD 935) for all migrants who have resided in Japan for at least 3 months, interest-free loan to small and big enterprise, free labor advice, etc. [49]. At the same time, participants seemed quite dissatisfied with the Nepal embassy in Japan and the Nepalese government for their lack of effort to address the needs of Nepalese migrants living in Japan. While participants were aware of the aid proffered by the Japanese authorities, they also noted various needs and suggested solutions for those needs, including (a) creating an easy and accessible scheme to return to Nepal, (b) offering financial support to survive, including helping with students’ loans, (c) disseminating reliable and clear information about COVID-19 testing and treatment in the Nepalese language, (d) establishing help desks to support concerns and queries (e.g., employment and/or visa information).

*M10: The Japanese government has planned a couple of schemes designed equally for migrants and Japanese citizens … . I think this is an exemplary step as our [Nepalese] government is not able to help us in any way.*

*F6: Even my children are receiving extra incentives from the government in order to mitigate the consequences of the pandemic. Facilities provided by the Japanese government amid COVID-19 must be appreciated. My only concern is that I had to face problems getting reliable information and testing for COVID-19, which should be addressed by the Japanese government.*

While some suggested that the sole responsibility is on the Japanese government and the companies they work for (because they work here and pay tax to the government), others attributed it to the Nepal embassy in Tokyo as the embassy is supposed to look after and provide help and assistance to all Nepalese living in Japan.

*M8: I think the Nepal Government cannot help Nepalese in Japan. Nepal is already dealing with issues in the country. If we were a citizen of a high-income country, we would not have come to Japan to work. I think the Japanese government should help as we are working in this country, paying taxes, and serving this country.*

*F3: [The] company where we work, and the city officials should be responsible. As we have been working for the company wholeheartedly and with complete dedication, it is their responsibility to help us (their employees) to keep us safe and secure in this unexpected scenario.*

*M7: As the Embassy of Nepal is the guardian of the Nepalese in Japan, they are the main responsible authority. They should be responsible for taking care of their citizens in a foreign nation. The embassy should make a help desk to listen to and address the concern of Nepalese migrants living in a foreign country.*

However, participants unanimously supported the need for multisectoral collaboration among the Nepal government, the Nepal embassy in Japan, the working organization, and the Japanese government to address problems faced by Nepalese living in Japan holistically.

*F1: Everyone (the Nepal embassy, Nepal government, and the Japanese government) should work together to help migrants.*

*M10: The current situation is totally unexpected, and we cannot rely on a particular government or institute for help. Rather, it should be systematic, and help should be provided by enhancing coordination between the two countries, in a way that no one is affected, and it would be sustainable.*

Despite numerous challenges faced by the Japanese Government in 2020, the participants expressed appreciation of the actions taken by the Japanese authorities and the manner in which they have tried to help Nepalese living in Japan. Overall, they have highlighted the need for a holistic approach to address the needs and concerns of the Nepalese living in Japan, which requires a collaborative and coherent approach from both countries.

## Discussion

The current COVID-19 pandemic has been the greatest contemporary global health, economic, and social challenge for humankind [50–52]. Our study was able to highlight the added and specific challenges of this crisis among international migrants (Nepalese citizens in Japan) owing to less stable employment conditions, lack of social support to deal with the emotional stress associated with pandemic-related crises, lack of social safety networks to rely upon in case of COVID-19 infection, problems of adjusting to life in Japan, obligations to send money to their families in Nepal, difficulty in accessing the available services and information due to the language barrier in the host country, and lack of effective governmental support/facilitation from Nepal. These all had direct negative consequences on their self-reported mental health and partly to their physical health, and these all could further prevent them from health and information-seeking opportunities and actions which have already been found to be perilously low among migrant populations globally [41, 53, 54].

Although a direct negative impact with respect to a decrease in income was seen among almost all participants, Nepalese with student visas experienced more severe challenges. These participants faced difficulties in paying rent, tuition fees, and even difficulty satisfying the needs of daily life. These findings are aligned with the study conducted among 9872 students in the United Kingdom where around 70% of students were worried about their ability to pay rent and other bills [55]. Similarly, around 500,000 migrant workers from Paraguay and Bolivia have lost their jobs and were struggling to survive in the host country [56]. Students in our study were even more vulnerable as they were relatively new to Japan and struggling to deal with transitions such as academic challenges, social isolation, and cultural adjustment. Furthermore, the new reality of COVID-19 has exacerbated their situation such that they were questioning their sustainability in a foreign nation. Insufficient command of the Japanese language might be another major stressor as they could not fully comprehend and utilize the information provided by the government as revealed in sub-theme 1.1 (Worried feelings and thoughts). Previous studies have also highlighted how linguistic problems pose barriers to accessing health services and acquiring health-related information among migrants populations [41, 57, 58]. Furthermore, students in this study highlighted the fact that they had to borrow heavy loans (with high-interest rates) for processing and tuition fees while coming to Japan, which they expect to return after working in Japan. As revealed in a previous study, a Nepalese who wants to work as a restaurant cook in Japan had to pay 1.5 million Nepali rupees (US$15,000) to a restaurant owner for sponsorship to enter Japan on a work visa [39]. Similarly, it costs nearly 1,466,000 yen ($14,054) to complete a two-year course at a Japanese language school, which is often paid in advance by loans [59]. Participants with high financial responsibility however are facing difficulty sustaining their daily living. So, it is likely for them to experience anxiety and stress, as a prominent finding in accordance with the international literature [60, 61].

Another affected participant group in our study appeared to be interviewees working in the restaurant sector or owning a business in that sector. In Japan, many Nepalese migrants are businesspersons who operate restaurants and/or work in a restaurant as a chef or as service staff [39]. In the last few years, Nepalese restaurants have been struggling due to high competition in the market [39]. Now, the sudden appearance of COVID-19 has a direct impact, further threatening the viability of these businesses. Interviewees in our study mentioned that they are heavily dependent on take-out and home delivery orders, as mentioned in theme 3 (Financial hardship). Loan facilities provided by the Japanese government to all businessmen have been a great relief, for now. However, the question about sustainability remains. Participants in this study were stressed thinking about their future and some of them were having doubts about whether it would be possible to carry on their businesses. These findings cohere with a study where restaurateurs mentioned their business’ probability of survival to be 72% if a crisis lasts for 1 month, 30% for 4 months, and 15% if it lasts for 6 months [62].

Despite numerous consequences associated with the economic problem, this is not the only issue facing this cohort. Information overload, perceived susceptibility to the virus, inability to access proper treatment, and lack of social support were major challenges for the participants in this study. These are all making them prone to different health problems. As introduced under sub-theme 1.1 (Worried feelings and thoughts), negative emotions like anxiety, stress, and overthinking were highly predominant among participants in this study. Information overload and fake news was found to be one of the sources of stress and perceived susceptibility of COVID-19 among participants in this study. There was a constant bombardment of frightening statistics about infected cases, death tolls, and unavailability of vaccines for COVID-19 in different information portals, particularly in online media. The growing tide of false, inflammatory, and misleading information was responsible for heightened anxiety among people. A study conducted in China also reported that more than 80% of participants were frequently exposed to social media and this exposure was associated with higher odds of anxiety and depression amid the COVID-19 pandemic [63]. While social media has a strong influence on the public and plays an important role in disseminating public health information, caution should be taken to prevent the spread of fake news that unnecessarily creates unfounded fear and harm to both the mental and physical health of the people.

Furthermore, studies have pointed out that social relationships typically exert a positive effect on immigrant health, such that social support from family and friends may serve as indicators of optimal levels of both emotional and mental health [64]. As highlighted in the sub-theme 1.1 (Worried feelings and thoughts), social support seems more crucial in the recent pandemic where migrants are facing a double burden of economic and mental problems. The perceived threat of disease coupled with a lack of social support was emotionally draining for the participants. Similar to the many incidences around the world where people are separated from loved ones due to travel restrictions [65, 66], participants in this study expressed dissatisfaction for being away from children and not being able to go back to the country.

Stigma and discrimination in the host country coupled with a lack of social support could be a major stressing factor for migrant residents in any host country during any major crisis. Fortunately, none of the participants in this study personally experienced any direct COVID-19-related stigma/discrimination as mentioned in theme 5 (Reflection on discrimination). However, they have recounted discrimination experienced by their relatives/friends in their daily lives. This aligns with the study findings conducted by the Ministry of Justice, Japan, where 40% of participants mentioned being affected by discrimination while trying to find accommodation; similarly, problems in workplaces, such as negative verbal remarks, employment being denied because they were foreigner, being paid less than their Japanese counterparts for similar work, etc. [33]. Likewise, multiple cases of discrimination against migrants have been reported in Japan during this pandemic, particularly from Chinese and Korean migrants. This includes receiving anonymous letters demanding that the Chinese should leave Japan, feeling neglected in certain municipalities for being Korean, etc. [67]. Moreover, an online survey conducted by Fukuoka Now (a local news magazine) among 413 foreign residents revealed that 18.2% of participants experienced some kind of COVID-19-related discrimination in Japan [68]. Citizens from a country with an initial high COVID-19 caseload might have been the main subjects for stigma as seen in other countries [69, 70]. Similarly, a study has highlighted the mixed attitude of Japanese towards migrants coming to Japan [71]. There is a need for in-depth exploration of these sensitive issues, which remain understudied in the Japanese context.

As evident from theme 6 (Reflections of existing support and expectations of support provision), the perception of participants towards non-governmental organizations was rather negative. Although the role of the local and non-governmental organizations is vital particularly during a pandemic or natural disaster, nevertheless, their accountability and transparency have been frequently questioned [72]. The reason for mistrust could be due to previous negative experiences or a lack of sufficient information about the contribution made by those organizations. Another reason might be migrants themselves are not actively searching and seeking facilities that are already available for them [73]. However, the findings of our study highlight that different local and non-governmental organizations should try to first develop trust among local people and disseminate information among them through different, appropriate media rather than just relying on a social media platform [46]. Any help provided by those organizations can make a huge impact on the life of vulnerable populations.

While the role of local and non-governmental organizations is important, the major contribution should be made by authorized governmental bodies. As mentioned by the participants in this study, multisectoral collaboration among the Japanese government, an employing organization/company, the Nepalese government, and the Nepalese Embassy in Japan, could be crucial. The research participants would welcome a collective, integrated response with shared responsibility across relevant authorities (as illustrated in theme 6: Reflections of existing support and expectations of support provision) for helping to meet the needs and concerns of the migrant community. Globally, countries are working to address the needs of their immigrants in this pandemic, for example ensuring migrant workers are included in national social protection measures, equal access to the health and related services as their citizens, or providing free screening and tests for non-citizens [32]. The Japanese government has made efforts to mitigate the impact of COVID-19 on international migrants, which was also mentioned by the participants in this study. However, as explained by the participants, information and easy accessibility of testing and treatment are concerns that need to be addressed. Language has become the topmost barrier in Japan that could make it difficult for migrants to access available health and needed services also. Unlike some countries, Japan does not have a legal framework that warrants the need for language interpreters particularly in hospital settings [74]. Furthermore, as suggested by the International Labor Organization, employer organizations should advocate for the rights of migrants workers, such as an extension of visa and work permit, and the provision of health services and social assistance regardless of their migration status [75]. Another important role could be played by the embassy, as they are considered responsible for addressing the concerns and safeguarding their citizens living abroad. Participants of this study expressed dissatisfaction with the Nepal embassy in Japan, and the Government of Nepal, for not being able to address the problem of migrants effectively. For now, as suggested by the participants, first, we argue that it would be useful for the embassy to evaluate needs locally rather than just drawing an understanding from those who seek help officially, who may represent a minority within the community. Second, migrant workers might be more vulnerable in a crisis situation like the COVID-19 pandemic, given the underlining inequalities and social factors surrounding their experience. As such, support for Nepalese citizen in Japan should be provided on a priority basis coordinating with the government of Japan and Nepal. Third, arranging affordable and accessible travel flights for those who are desperately in need is seen as an important concern within the community and will alleviate the sense of helplessness and being stranded in a foreign place. Finally and critically, it is important to create a service to collect and listen to the voices of the community itself, through a helpline or other tool.

There are some limitations to this study. First, this study was conducted among Nepalese migrants residing in Japan during the period of the study only: our findings may not be generalizable to other migrant populations living in Japan or elsewhere. The sample size of the study was limited; however, a saturation of the response was taken into account. Third, our analysis was solely based on the participants’ responses; while these lived experiences are undeniably important, further evidence needs to be generated to determine policy options and implementations around this issue. Lastly, the interviewer was also a Nepali living in Japan, which might have caused biases. However, such biases were minimized by the research team reviewing the interpretation of data.

The experiences of migrants are contextual and therefore the findings cannot be widely generalized to other populations in other places. Nevertheless, the findings of this study will add information to the ongoing debate with regard to what policies and best practices can be introduced and policed to help protect minority groups in crisis situations in countries around the world.

## Conclusion

Our study provided an understanding of challenges faced by the non-Japanese Nepalese community members in Japan amid the COVID-19 pandemic as identified by individuals within the community. Immediate challenges faced by participants relate to financial shortfalls arising as a result of the pandemic, including difficulty in paying rent and tuition fees, loss of wages, and difficulties in conducting business. In health terms, fear of getting infected, concerns about the family, and lack of information on testing/treatment were paramount. Anxiety and fear were exacerbated by visa-related problems, travel restrictions, and possible legal matters. Furthermore, our study was able to shed light on factors such as lack of social support, the impact of the language barrier, occupational instability, family obligations, and other factors that inequitably affect the health and livelihoods of non-Japanese community members in Japan and which exacerbate their vulnerability in a crisis such as the on-going COVID-19 pandemic. Overall, our study offered helpful insights for researchers, practitioners, advocates, and policymakers to provide targeted and integrated support in order to mitigate these health, financial and social impacts on the wellness and lives of Nepalese migrants living in Japan during crises like COVID-19 pandemic.

## Supplementary Information

**Additional file 1.** Interview Guide (English). This file contains the interview guide developed and used in this study.

## Data Availability

The datasets used and/or analyzed during the current study are available from the corresponding author upon reasonable request.
