# Supplementary material for: COVID-19: challenges faced by Nepalese migrants living in Japan
Source: BMC Public Health. 2021 Apr 19;21:752. doi: 10.1186/s12889-021-10796-8 (PMC8054259; doi:10.1186/s12889-021-10796-8)
Supplement: Supplementary file 1 — Additional file 1. Interview Guide (English). This file contains the interview guide developed and used in this study. [file 12889_2021_10796_MOESM1_ESM.docx]

**Interview guide**

Section1: Questions on concerns and needs

1. Could you tell me a little bit about how you feel at the moment, in terms of your health? Do you feel that your level of physical health has changed since the pandemic period? In what ways?
   1. So, we talked a bit about your physical health, do you see changes in respect to your mental health?
2. What are you worried about in terms of the current period?
   1. Do you have any other concerns? For example, some people are really worried about their finances in the current context. Please think about any area of your life, e.g, health, finance, work, family, social life.
3. Do you think there is anything particularly challenging about being a (Nepalese) migrant in the current context? Are there any ways in which the pandemic has an impact on you that you think are specifically an outcome of being a migrant?
   1. When you talk about [x] was it something that has happened to you or something that’s happened to someone you know or just heard about somewhere?
   2. Do you think that [y] is specific to the pandemic, or is it something that was also happening to some extent before the pandemic period?
   3. What do you think is the cause of these sorts of experiences?
4. So we’ve talked about [x,y,z,] concerns. Who do you think could be helpful in managing these concerns? What types of supports or interventions might be useful to you?
   1. Do you think [Japan government, Nepal embassy, NRNA Japan] would be the best place to help in this way? Why?
   2. Are there any support services out there already that you’ve either accessed or heard about?
   3. Do you think that [these organizations] are listening to your concerns at the moment, or do you feel that the Nepalese community needs further assistance?

Section2: Background Information

Demographics:

- 1. Age
  2. Gender
  3. Length of time in Japan
  4. Immigration/Visa status
  5. Employment status
  6. Financial status (Monthly income)
  7. Education level – number of years in school?
  8. Japanese language skill
